# Land use and soil type determine the presence of the pathogen *Burkholderia pseudomallei* in tropical rivers

**DOI:** 10.1007/s11356-015-5943-z

**Published:** 2016-01-13

**Authors:** Olivier Ribolzi, Emma Rochelle-Newall, Sabine Dittrich, Yves Auda, Paul N. Newton, Sayaphet Rattanavong, Michael Knappik, Bounsamai Soulileuth, Oloth Sengtaheuanghoung, David A. B. Dance, Alain Pierret

**Affiliations:** Géosciences Environnement Toulouse (GET), UMR 5563, (IRD, CNRS, UPS), Université de Toulouse, UPS (OMP), CNRS, Toulouse, France; iEES-Paris (IRD-UPMC-CNRS-INRA-UDD-UPEC), Sorbonne Universités, UPMC Univ Paris 06, Institut de Recherche pour le Développement (IRD), case 23, 4 place Jussieu, Paris cedex, 75252 France; Lao-Oxford-Mahosot Hospital-Wellcome Trust Research Unit, Microbiology Laboratory, Mahosot Hospital, Vientiane, Lao People’s Democratic Republic; Centre for Tropical Medicine and Global Health, Nuffield Department of Medicine, University of Oxford, Oxford, UK; Institute of Ecology and Environmental Science—Paris, Institut de Recherche pour le Développement (IRD), Vientiane, Lao People’s Democratic Republic; Department of Agricultural Land Management (DALaM), P.O. Box 4199, Ban Nogviengkham, Xaythany District, Vientiane, Lao People’s Democratic Republic

**Keywords:** Melioidosis, Acrisols, Ferralsols, Turbidity of river water, South East Asia, Partial least squares discriminant analysis (PLSDA), Geographical information system (GIS), Watershed

## Abstract

*Burkholderia pseudomallei* is the bacterium that causes melioidosis in humans. While *B. pseudomallei* is known to be endemic in South East Asia (SEA), the occurrence of the disease in other parts of the tropics points towards a potentially large global distribution. We investigated the environmental factors that influence the presence (and absence) of *B. pseudomallei* in a tropical watershed in SEA. Our main objective was to determine whether there is a link between the presence of the organism in the hydrographic network and the upstream soil and land-use type. The presence of *B. pseudomallei* was determined using a specific quantitative real-time PCR assay following enrichment culture. Land use, soil, geomorphology, and environmental data were then analyzed using partial least squares discriminant analysis (PLSDA) to compare the *B. pseudomallei* positive and negative sites. Soil type in the surrounding catchment and turbidity had a strong positive influence on the presence (acrisols and luvisols) or absence (ferralsols) of *B. pseudomallei*. Given the strong apparent links between soil characteristics, water turbidity, and the presence/absence of *B. pseudomallei*, actions to raise public awareness about factors increasing the risk of exposure should be undertaken in order to reduce the incidence of melioidosis in regions of endemicity.

## Introduction

*Burkholderia pseudomallei* is an environmental bacterium that is pathogenic to humans. It causes melioidosis, a disease that is thought to be responsible for a substantial yet undetermined number of fatalities every year in the tropical belt (Cheng and Currie 2005; Dance 1991). The pathogen is endemic in South East Asia (SEA) (Buisson et al. 2015; Limmathurotsakul et al. 2014) and in Northern Australia, where the bacterium has been detected in drinking water (Currie et al. 2001; Draper et al. 2010). However, the occurrence of cases in other tropical locations points towards a potentially large global distribution. Cases have also been reported in non-tropical areas, for example North America, although these cases are usually related to exposure in other regions, as for example in the case of veterans believed to have been infected during the Vietnam War (Ngauy et al. 2005). Nevertheless, the largest proportion of cases by far occurs in low- and middle-income countries or emerging economies such as Laos and Thailand (Palasatien et al. 2008; Sermswan et al. 2001; Wuthiekanun et al. 2009). However, and despite the pathogenic nature of this environmental bacterium and its likely high global prevalence (Currie et al. 2008), little is known about the factors that control its distribution in the environment.

Several reports indicate that a range of environmental factors might influence the distribution of *B. pseudomallei*. Corkeron et al. (2010) observed that soil type, geomorphic position, and drainage were related to the distribution of clinical cases of melioidosis in the Townsville region of Northern Australia. These authors found that cases were associated with two distinct geomorphic and soil types. The first was piedmont slopes adjacent to granitic hill and mountain slopes with Kandosols characterized by dark gray-brown loamy sand to silty loam A horizon, grading into dark red or yellow sandy clay loam to sandy clay subsoils. The second was Pleistocene floodplains, levees and channel-fill where soils typically graded from acidic to alkaline at depth. These soils were also poorly draining due to a shallow impermeable B horizon, similar to what is observed in paddy fields. Such findings were further supported by Inglis (2010) who underlined the importance of geomorphology and soil type in the potential emergence of pathogenic bacterial species and concluded that some soils must be regarded as a potential health hazard.

Data from epidemiological and field studies have highlighted a potential link between land use and the presence of *B. pseudomallei* as many cases of human infection have been associated with working in paddy fields (Limmathurotsakul et al. 2010; Rattanavong et al. 2011; Vongphayloth et al. 2012). A survey of transmission modes showed that patients with *B. pseudomallei* infection were most often individuals who had experienced flooding and had walked barefoot on soil, both of which can be considered as factors increasing the risk of exposure to *B. pseudomallei* (Su et al. 2011). Consequently, it can be hypothesized that paddies increase the risk of contamination not just because the pathogen is more abundant in such environments but also because people are working barefoot in paddy fields and regularly sustain minor injuries and abrasions. Moreover, given the spatial heterogeneity of the geographic distribution of *B. pseudomallei*, it is clear that further studies are needed to investigate its potential climatic, edaphic, and biological determinants (Rattanavong et al. 2011).

Other work has pointed towards the links between iron and the presence of *B. pseudomallei*. Iron is the fourth most common element in the Earth’s crust, and the vast majority is distributed as iron minerals in soils and sediments (Stumm and Sulzberger 1992). Baker et al. (2011) reported that higher *B. pseudomallei* abundance was found in groundwater seepages in a region with soils with high iron oxide contents. Similarly, Draper et al. (2010), working in Northern Australia, found that *B. pseudomallei* was associated with soft, acidic bore water of low salinity and high iron levels. It therefore seems that iron availability could potentially play a role in controlling *B. pseudomallei* in the environment. Ferric iron (Fe^3+^) and ferrous iron (Fe^2+^) are the main redox states found in the environment and play pivotal roles in global biogeochemistry as part of the microbial and abiotic redox cycling of this element (Melton et al. 2014). Furthermore, microbial Fe^3+^ reduction is one of the most significant electron sinks for the oxidation of organic compounds under anoxic conditions prevailing in natural ecosystems (Hori et al. 2015; Melton et al. 2014). In soils, the concentration of available dissolved iron is dependent on depth, soil salinity, and on agricultural practices. Indeed, Fe^2+^ can represent up to 90 % of total dissolved iron in rice paddies in Thailand (Saejiew et al. 2004). Moreover, the addition of organic matter (e.g., compost or manure) which is common in rice production systems can dramatically increase the solubilization of iron (Grunberger 2015), which may, in turn, favor microbial species with mechanisms that allow them to take advantage of this iron.

Land use in rural tropical areas is rapidly changing with a general switch from natural forests to perennial or annual cash crops. One immediate consequence of this shifting land use is to increase soil erosion and turbidity in streams and rivers (Valentin et al. 2008). Indeed, soil erosion can be particularly important in tropical areas where rainfall events are generally intense and erosion is high (Valentin et al. 2008). Although the implications of soil erosion on soil fertility and the loss of biodiversity are intuitive, it is less evident how soil erosion affects the transport of soil bacteria, including pathogens, from soils to downstream hydrographic networks.

Land use and overland flow play an integral role in the transfer of bacteria on and in soils to downstream aquatic systems in tropical, rural areas (Causse et al. 2015; Ribolzi et al. 2015). Indeed, *B. pseudomallei* is naturally found in some tropical, rural environments and the potential for transport and contamination of a non-contaminated site should not be ignored. In both soils and aquatic systems, bacteria are often associated with particles in the environment and given that *B. pseudomallei* is a hydrotelluric bacterium, we hypothesized that turbidity will be an important factor in determining the presence of *B. pseudomallei*.

This work aimed to investigate the environmental factors potentially influencing the presence of *B. pseudomallei* in stream and river samples. Our main objective was to establish a link between the presence of this bacterium (*B. pseudomallei*) in the hydrographic network and land use in a tropical drainage network where these bacteria have already been identified (Vongphayloth et al. 2012) and the upstream soil and land-use type. The secondary objective was to determine if there was a relationship between these pathogenic bacteria and turbidity in the hydrographical network.

## Materials and methods

### Sample collection

**Fig. 1:**
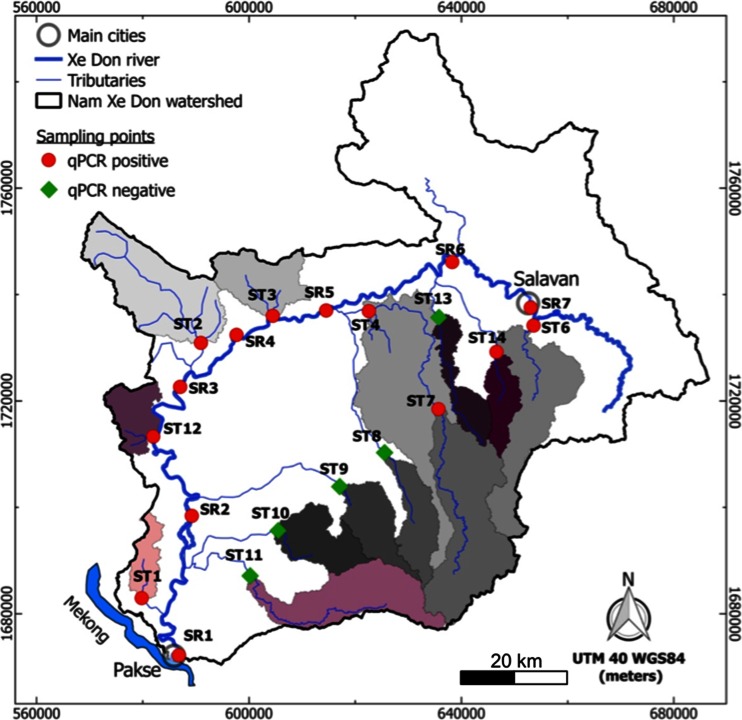
Nam Xe Don watershed and the sub-catchments sampled for the survey (*gray* and *purple* areas); water sampling stations along the Xe Don main stream (SR) and its tributaries (ST); presence (*red dots*) or absence (*green diamonds*) of *B. pseudomallei* determined using a specific quantitative real-time PCR assay

The study area is located in Southern Laos (SEA) in the Provinces of Saravan and Pakxe (Fig. 1). Water samples (600 ml) were collected from 20 sites along the Nam Xe Don (Xe Don River) and its main tributaries in June 2013. Seven samples were collected along the main river path (denoted SR; Fig. 1) and 13 from the tributaries (denoted ST; Fig. 1). Samples were stored in a cooler with ice packs before gentle vacuum filtration (3.0 and 0.2 μm, cellulose acetate Sartorius™ membrane filters) within 8 h (Knappik et al. 2015). Temperature (°C), conductivity (EC; μS cm^−1^), pH, dissolved oxygen (DO; %), redox potential (ORP; mV), and turbidity (NTU) were measured in situ with an YSI-556 probe and an EUTECH instrument, respectively.

### Laboratory analysis

The presence or absence of *B. pseudomallei* was determined on both the 3.0- and 0.2-μm filters using the method previously described by Knappik et al. (2015). Briefly, filters were enriched overnight in Ashdown’s broth and DNA extracted using a commercial kit (PowerSoil DNA extraction, MolBio, UK). Subsequently, a 115 bp (orf2) of the type III secretion system gene cluster of *B. pseudomallei* was amplified using a specific quantitative real-time PCR (qPCR) assay (Novak et al. 2006). Positive controls and none template controls, which always showed the expected result, were included in each run (Knappik et al. 2015).

### Geographical analysis

**Fig. 2:**
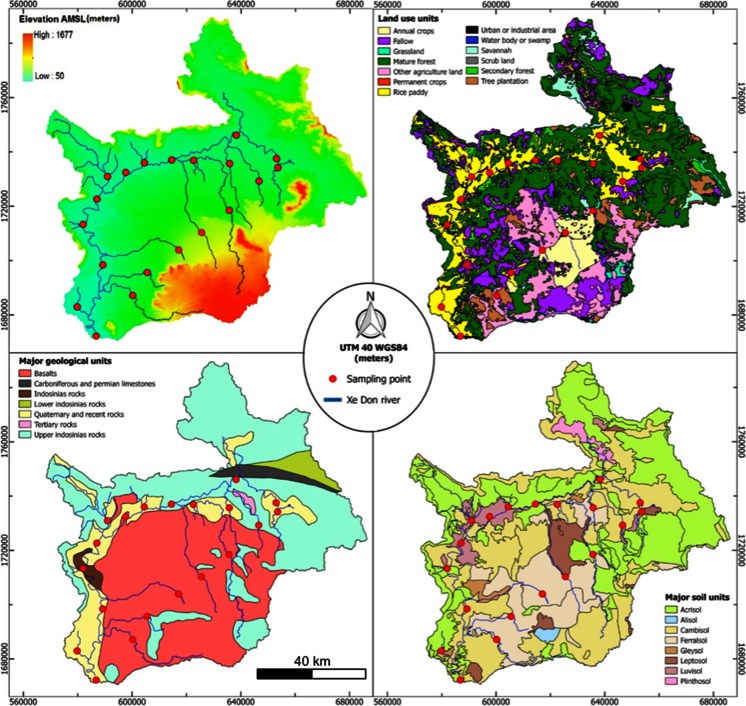
Mean geographical characteristics of the Nam Xe Don watershed: geomorphological features (Digital Elevation Model); land use units; geological units; major soil type units

The 30-m resolution digital elevation model (DEM) “SRTM” (http://reverb.echo.nasa.gov/reverb/) was used to draw the elevation map (Fig. 2a) in the QGIS 2.8 software (https://www.qgis.org/en/site/forusers/). The model has a mean accuracy of 20 and 16 m in the east–west and north–south directions, respectively. Based on this DEM and the geographical location of the sampling points, the drainage areas were then determined using the Geographic Resources Analysis Support System GRASS 7.0 (http://grass.osgeo.org/download/software). Perimeter, median slope, and median of the average elevation of each of the drainage areas upstream of the sampling points were also computed from the DEM (Fig. 1).

### Land use

Land use in the drainage area was constructed using the data provided by the DALaM (Department of Agriculture Land Management) of Laos (Fig. 2b). The 32 different categories of land use (e.g., annual crops, forests villages, etc.) were grouped into 13 main categories (e.g., annual crops, permanent crops, fallow, rice paddies, other agricultural land, secondary forest, mature forest, scrub lands, savannah, tree plantation, grasslands, water bodies or swamps, urban or industrial areas) with a clear difference between uplands (% agricultural lands, tree plantations, and annual crops) and the lowlands (mostly paddy fields and mature and secondary forests and fallow).

### Geology and soils

The geological map (Fig. 2c) was constructed using a geological map of Laos (MRC). The Nam Xe Don watershed comprised six geological categories: basalts, Carboniferous and Permian limestones, Lower Indosinias rocks, Upper Indosinias rocks, Tertiary rocks and Quaternary and recent rocks. The soil map was constructed using the data of the Mekong River Commission (MRC (MRC)). Following the FAO (FAO 1998) classification, the map is made up of 8 categories (Fig. 2d): acrisols, alisols, cambisols, ferralsols, gleysols, leptosols, luvisols, plinthsols. The percentage area of each soil, geology, and land use characteristic was then estimated in each drainage area using the Geographical Information System (GIS) database.

### Statistical analysis

The PLSDA analysis was conducted on the 0.2-μm fraction as this fraction represents the total fraction (i.e., included both free bacteria and those attached to particles). Given that the 7 stations along the mainstream of the Xe Don River (Fig. 1) are all positive for *B. pseudomallei* and are influenced by confounding variables because of their integrative nature (i.e., their large drainage area covers almost all soil types and land use), we chose to limit the data analysis to the 13 stations located in the tributaries in order to highlight the most discriminant variables associated with *B. pseudomallei* positive or negative cases. With the exception of the station 4 (ST4) that is located downstream from station 7 (ST7), the remaining 12 catchments corresponding to these points have similar areas but exhibit contrasting morphopedological, physico-chemical, and land use characteristics (Fig. 2).

As the dataset has a higher number of variables than individuals (sample points) and as some of the variables are probably correlated, the partial least squares discriminant analysis (PLSDA) was used to discriminate the two groups of individuals (*B. pseudomallei* positive and negative). PLSDA is an alternative to factorial analyses, but unlike factorial analyses, PLSDA is not based on the scheme of duality, which is unsuitable given the structure of our dataset (Benzecri 1973; Brereton and Lloyd 2014; Cailliez and Pages 1976; Pages et al. 1979). PLSDA is based on an iterative algorithm as described by Collins (2010) and Brereton and Lloyd (Brereton and Lloyd 2014). The importance of each projected variable is estimated by the variable importance in the projection number (VIP; a value between 0 and 2). Values below 1 are considered to be unimportant in the analysis. The ADE4 package was used to perform the graphical outputs (score graph of the VIP values and a correlation circle of the variables) (Hervé 2013; Thioulouse et al. 1997). In order to test the appropriateness of the PLSDA method and to validate the model, a cross-validation was performed to address potential concerns of model over-fitting (Westerhuis et al. 2008). Given the small number of individuals involved in the analysis (13 individuals), a simplified procedure using two folds, i.e., a learning fold and a testing fold, was conducted. The learning and testing folds were set at 3 and 10, respectively. As recommended by Szymanska et al. (2012), the number of misclassifications (NMC) was used in the cross-validation test. A permutation procedure of the class label (qPCR positive, qPCR negative) of the learning fold individuals was performed in order to obtain a set of NMC. The arithmetic mean of these NMC was then used as a criterion to estimate PLSDA quality. The observed criterion was compared to the simulated theoretical distribution, and the *P* value was calculated following the formula recommended by Szymanska et al. (2012).

## Results

### Physico-chemical parameters

Temperature ranged between 24 and 28 °C with the lowest temperatures measured at the sampling points along the tributaries downstream of the Boloven plateau (Table 1). EC varied between 22 and 112 μScm^−1^ without following any clear spatial pattern. Similarly, DO varied between 54 and 92 % saturation with no spatial pattern. In contrast, redox varied by over a factor of 10 with the lowest values (28 mV) at ST13 and the highest in ST2 (262 mV), and pH varied by two pH units (5.27–7.82) with the lowest values observed at ST2. Turbidity also varied widely (18.2–727; CV 74 %). The lowest turbidity values were found in the tributaries downstream of the Boloven plateau, and the highest were found in Xe Don River proper.

**Table 1:** Sample date (2013), geographical position (i.e., Easting and Northing in meters, UTM coordinates, WGS84 ellipsoid model) of the stations, and physico-chemical parameters of river water measured in the field

Station	Date	Easting	Northing	pH	T	EC	DO	ORP	Turbidity
					(°C)	(μS cm^−1^)	(mg l^−1^)	(mV)	(NTU)
SR1*	Jun 24	586795	1672195	6.32	25.3	30	80.3	107	727
SR2*	Jun 24	589227	1698402	6.64	25.7	27	68.6	99	542
SR3*	Jun 24	587038	1722644	6.42	25.9	24	54.4	120	446
SR4*	Jun 25	597648	1732416	7.22	26.9	45	89.6	86	362
SR5*	Jun 25	614528	1737011	7.41	26.7	48	92.4	52	271
SR6*	Jun 25	638258	1746117	7.28	26.9	55	85.2	66	177
SR7*	Jun 26	653049	1737542	7.1	26.6	100	76.8	98	313
ST1*	Jun 24	579802	1682945	6.75	28.2	22	92.2	96	319
ST2*	Jun 25	590938	1730920	5.27	28.2	24	80	262	585
ST3*	Jun 25	604481	1736008	7.07	28.2	26	78	85	404
ST4*	Jun 25	622572	1736890	7.41	26.9	68	71	50	56
ST6*	Jun 26	653585	1734185	7.74	26.2	62	83.3	69	237
ST7*	Jun 26	635686	1718471	7.63	23.9	40	89.3	59	54
ST8	Jun 26	625524	1710274	7.82	25.3	112	91.2	62	46
ST9	Jun 26	617082	1703896	7.39	24.4	30	86.9	51	74
ST10	Jun 25	605520	1695614	7.11	24.3	39	80.9	55	18
ST11	Jun 25	600217	1687124	7.32	23.9	25	87	90	26
ST12*	Jun 24	581965	1713306	6.53	26.4	28	57	122	422
ST13	Jun 25	635772	1735692	7.57	27.2	86	84.5	28	118
ST14*	Jun 26	646610	1729230	7.49	27.2	70	84.1	77	417

The stations in the tributaries (Stations ST1 – 14) were used in the PLSDA analyses.* denotes that the station was positive for *B. pseudomallei*

pH, *T* temperature, *EC* electrical conductivity, *DO* dissolved oxygen, *ORP* oxydo-reduction potential, turbidity

### *B. pseudomallei*

Of the 20 samples analyzed, 15 were positive for the presence of *B. pseudomallei* (Fig. 1). Of the five samples that were negative for *B. pseudomallei*, four were sampled in tributaries from the Boloven plateau (Fig. 1; ST8 to ST11). The only exception was ST13 that was not from the Boloven plateau.

**Fig. 3:**
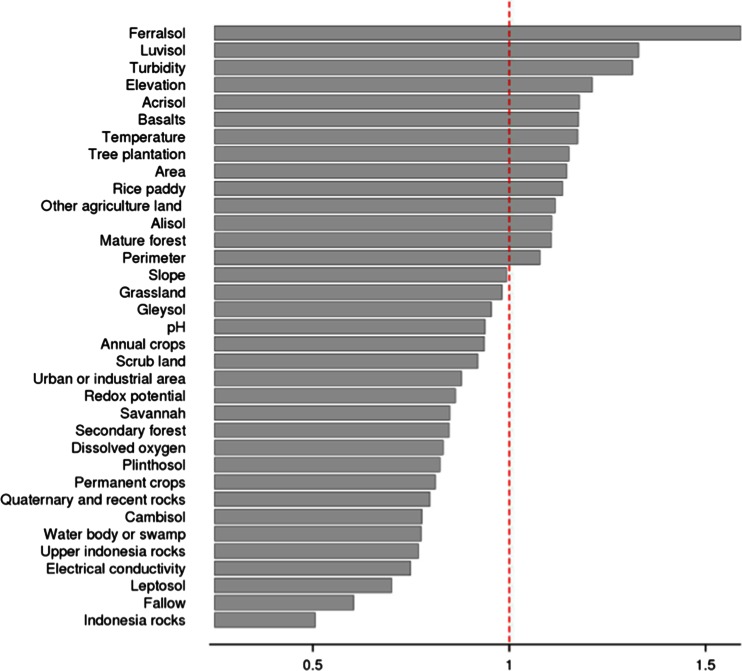
Variable importance in the projection (VIP) for the tributaries (*n* = 13). Values less than 1 are considered to be marginally influential. The *dotted vertical line* represents the cutoff at a VIP of 1

**Fig. 4:**
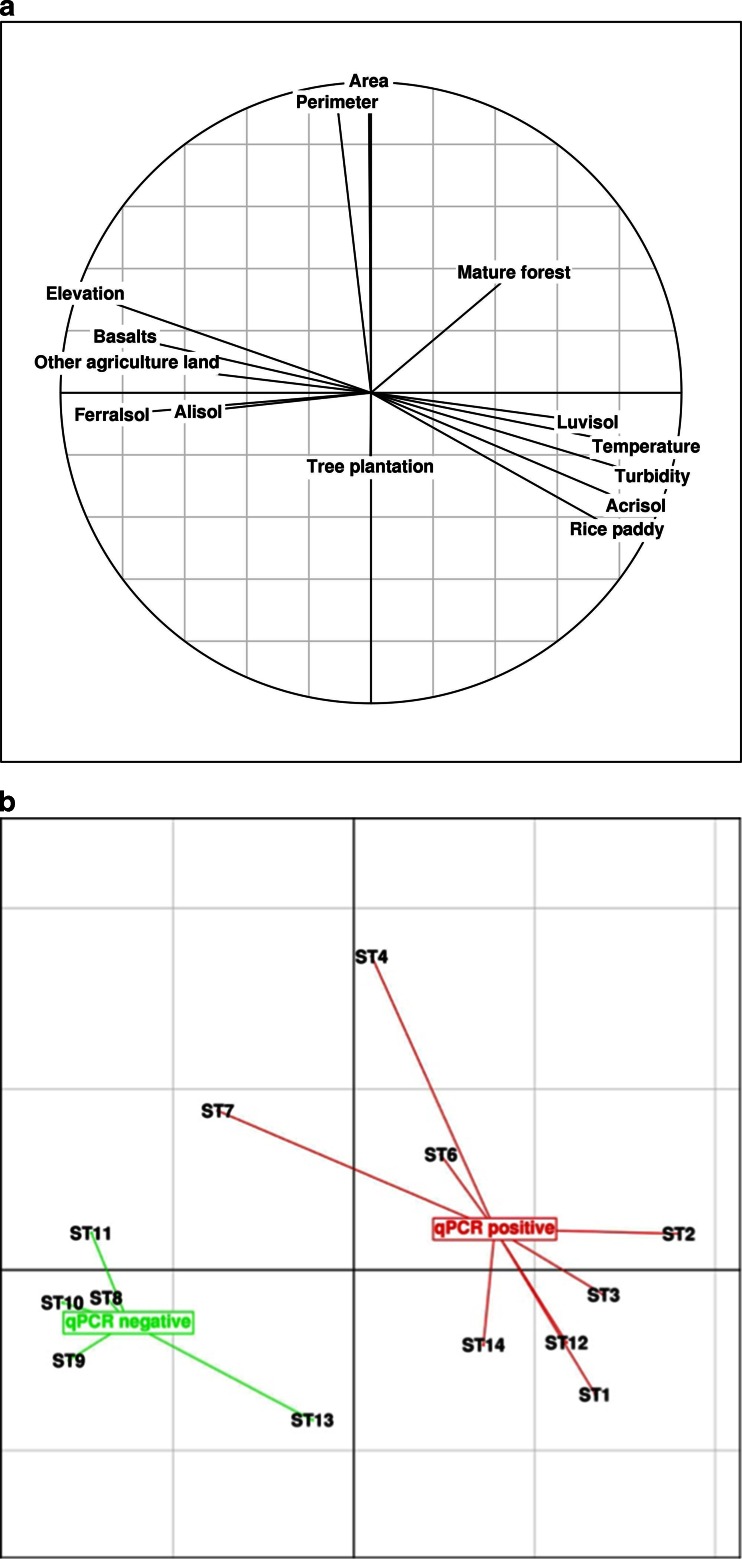
Correlation circle and factorial map from the first two axes for the main variables (VIP > 1) from partial least square analysis (PLSDA). **a** Correlation circle; **b** Factorial map. qPCR positive and negative samples are reported in *red* and *green*, respectively. Each sample is connected to the barycenter of each group of point

The ferralsols had the highest VIP number (>1.5) in the PLSDA (Fig. 3). This was followed by, in decreasing order, turbidity > luvisols > elevation > acrisols > basalts > temperature > area > rice paddies. All of the other factors had a VIP below 1 and were therefore considered to be of marginal influence. The correlation circle and the factorial map are given in Fig. 4a, b. When only VIP values above 1 are considered, the first and second axes explain 93 and 5 % of the variance, respectively. The second axis corresponds to variables that are strongly related to the size of the drainage area (e.g., perimeter and area). There was a strong separation of the qPCR-positive and qPCR-negative values along the first axis with the barycenter of the two groups being located on either side of the second axis (Fig. 4b). The variables corresponding to this separation are easily identifiable in the correlation circle as they form two main groups separated along the first axis. The positive sites, located on the left side of the factorial map, were associated with turbidity, luvisols, acrisols, temperature, and the presence of rice paddies. In contrast, the negative sites, located on the right hand side of the factorial map were associated with ferralsols, elevation, basalts and to a lesser extent “other agricultural lands”. However, the separation along the second axis (drainage area) does not appear to be strongly associated with sample positivity. The cross-validation shows that the results obtained are robust and are not due to over-fitting (NMC arithmetic mean of 0.62; *P* value < 0.01).

## Discussion

Here, we present the results of an investigation into the environmental factors that influence the presence (and absence) of *B. pseudomallei* in rural, tropical watershed (Xe Don River, a main tributary of the Mekong River). We show that two of the principal factors controlling the presence or absence of this pathogenic bacterium are high turbidity (positive effect) and the presence of ferralsols (negative effect).

### Soil type and bioavailability of nutrients

The qPCR negative stations (ST8 to ST11 and ST13) were all strongly associated with the presence of ferralsols. Ferralsols are one of the 30 major soil groups of the World Reference Base for Soil Resources (FAO 1998). These soils have high porosity and permeability meaning that they are rarely anoxic. Paradoxically, although these soils have high particulate iron contents, dissolved bioavailable iron (Fe^2+^, Fe^3+^) is scarce. This is due to the permeability that enables renewal of the gases (particularly O_2_ and CO_2_) dissolved in the soil solution and involved in redox equilibrium that maintains iron in stable solid forms as oxides and oxy-hydroxides.

In contrast, the qPCR positive stations were positively associated with acrisol and luvisol soil types. Acrisols are also one of the 30 major soil groups of the World Reference Base for Soil Resources (FAO 1998). These soils are poorly draining due to the presence of a shallow impermeable B horizon, and as a consequence, they are often used for the cultivation of irrigated rice. However, the practice of flooding rice paddies means that water flow and gas renewal within the soil are drastically reduced. This in turn affects the physico-chemical conditions: decreasing pH (<4) and ORP and reducing metal oxides and oxy-hydroxides to their soluble, reduced form. Luvisols were also positively associated with the qPCR-positive sites. Among the luvisols, the gleyic class (gleyic luvisols) is by far the most dominant in the study area. It is therefore probable that in irrigated acrisols and gleyic luvisols, physico-chemical conditions (lower pH, etc.) favor the mobilization of iron (Stoops and Eswaran 1985) and other nutrients, which may confer an advantage to certain bacteria such as *B. pseudomallei* (Baker et al. 2015).

### Erosion, turbidity, and groundwater contribution

Within the positive samples, there was no difference between the 3- and 0.2-μm fractions, indicating that *B. pseudomallei* was always associated with the >3.0-μm fraction. We interpret this as being indicative of the high rates of particle attachment of these bacteria. One of the main implications of this result is that the stream transfer of *B. pseudomallei* is in the attached form, which is in agreement with the statistical analyses that showed the importance of turbidity as a main determinant variable of the presence of this pathogen. The relationship between *B. pseudomallei* and turbidity has also been observed in rural water supplies in Northern Australia (Draper et al. 2010).

Transport of *B. pseudomallei* from a primary reservoir source can be facilitated by groundwater seepages (Baker et al. 2011; Baker et al. 2015) and river and stream bank erosion preferentially occurs at points of groundwater seepage along rivers (Slide and Ochiai 2013). Such processes of soil and sediment mobilization during flood events results in the massive and simultaneous mobilization of contaminants and particle-bound bacteria such as *B. pseudomallei*. The causal links between groundwater movement, riverbank erosion, instability, and particle-bound contaminant transport may also partly explain why *B. pseudomallei* is associated with high turbidity levels.

### *B. pseudomallei* and iron

*B. pseudomallei* has been found in soft bore water with low pH, low salinity and high iron (Fe) levels. Iron is a major nutrient for microbial metabolism and as iron is a limiting nutrient in many habitats, *Burkholderia* spp. have evolved a wide range of strategies to overcome iron shortage and to ensure sufficient uptake. The most common systems rely on the secretion of low molecular weight, high affinity iron-binding siderophores (Guerinot 1994; Mathew et al. 2014; Neilands 1995) although some *Burkholderia* spp. are also capable of acquiring iron from hematite and ferritin (Kvitko et al. 2012; Whitby et al. 2006). It is therefore probable that in sites where iron is limiting, bacteria that possess such mechanisms to taking up iron when it is limiting will be at an advantage.

Iron can also be used as a chemical energy source during redox reactions between electron donors (such as Fe^2+^) and electron acceptors (Fe^3+^) in oxygen limiting conditions (Kato et al. 2012) similar to what occurs with nitrate and nitrite (e.g., Rodionov et al. 2005). Indeed, *B. pseudomallei* is a member of the beta-proteobacteria and is known to be capable of anaerobic respiration with NO_3_^−^ in oxygen limiting conditions such as that found in flooded, anoxic paddy soils. The reduction of Fe^3+^ oxides (or hydroxides) is one of the most important electron sinks for organic compound oxidation in natural environments. Recently, iron oxide reducing microorganisms in soils and sediments have been isolated (Ding et al. 2015; Hori et al. 2015), and although this work did not specifically focus on *B. pseudomallei*, the group to which this bacteria belongs, the beta-proteobacteria were found in the analyzed sequences.

### Land use

*B. pseudomallei* has previously been reported to be present in rice paddies in Thailand and Laos (Limmathurotsakul et al. 2010; Rattanavong et al. 2011). We also found that several of our positive points (e.g., ST2) were close to a rice paddy. We propose that this presence is an indirect consequence due to several factors. First, water saturation creates the specific physico-chemical environment (i.e., iron in a bioavailable form) that favors the presence of the bacterium. Second, the presence of high concentrations of organic matter that will further feedback to maintain this specific physico-chemical environment as well as supplying a source of organic matter for bacterial metabolism. The addition of organic matter to soils tends to increase anoxia, particularly so in irrigated rice fields (Supparattanapan et al. 2009). This results in a decrease in the redox potential concomitant with a dramatic increase in the concentrations of bioavailable Fe^2+^. As a consequence of the reduction of iron to the Fe^2+^ form and the consummation of protons that this process imposes, an increase of pH can occur (Kirk 2004). However, the precipitation of hydroxy-green rusts (Bourrié et al. 1999) tends to, in turn, stabilize the pH towards neutrality, thereby reducing the impacts of pH changes on the bacteria present. A further factor is linked to the downstream nature of paddy fields. These systems are often the receptacle for upstream erosion of soils but also of eroded microbial biodiversity. This can be particularly critical in the case of environmental bacteria that are pathogenic to man as paddy fields are also a site of human activity, which increases the chances of exposure.

Moreover, ST7 was positive, despite being close to the negative grouping on Fig. 4. This is partly due to a significant percentage of ferralsols (14 %), a low turbidity (54 NTU), and a low fraction of rice paddy (about 1 %). However, one third of the drainage area is composed of acrisols (positive effect) free of rice paddy but located on the right bank in direct contact with the river (Fig. 2). We propose that the presence of *B. pseudomallei* at this location is due to the contamination of the hydrological network from the adjacent acrisols zone. In other words, ST7 is potentially a weakly contaminated site, which is supported by the fact that qPCR measurements made on non-cultured samples (Table 2 of Knappik et al. (2015)) from this site were negative whereas they became positive after culturing. In contrast, ST13 which has a zone of ferralsols downstream is qPCR negative despite being adjacent to a paddy field. We propose that despite being adjacent to a paddy field (positive effect; Fig. 3), the presence of ferralsols, with a high negative effect, explains the overall absence of *B. pseudomallei* at this sample site. Therefore, we propose that paddy fields are more of an “aggravating factor” that will accentuate the anaerobic soil conditions rather than a determining factor of the presence of *B. pseudomallei*.

### Methodological approach

Limmathurotsakul et al. (2010) noted that the available information on the spatial distribution of *B. pseudomallei* is limited which renders the construction of an adequate sampling strategy that minimizes false negatives complicated. Indeed, these authors have shown that *B. pseudomallei* is spatially distributed at the plot-scale (Limmathurotsakul et al. 2010), rendering the collection of adequate soil samples difficult and uncertain, particularly if large areas need to be examined. This can be circumvented using a catchment-scale approach as it integrates the local variability of soil properties. Indeed, rivers can be viewed as being an integrator of water, solute, and solid fluxes in a catchment. We therefore chose to sample along the hydrographic network in order to establish a link between the presence and absence of bacteria at the sampling point and the characteristics of the upper drainage areas. This method is advantageous as the sampling of the water column provides an integrated estimate of the forcing factors upstream of the sampling point. By combining this information with other environmental parameters from the upper catchment, we can try to estimate the factors influencing the presence or absence of *B. pseudomallei* at a given site.

This work is also based on a set of 20 sample sites that were collected once, and obviously, this provides only a snapshot of what might be happening over longer time scales. This is particularly important as it is known that the Mekong tributaries exhibit considerable seasonality in terms of suspended particles (Shrestha et al. 2013) and hence in turbidity. In order to take this into account, we selected the month of June for this study because it is the beginning of the rainy season in Laos. We hypothesized that the sub-basins of the Nam Xe Don would have soils that were “sensitive” to erosion during this period which would result in more turbid flows and therefore a higher probability of contamination. Nevertheless, it is clear that a longer term study would be interesting if any seasonal patterns are to be discerned.

Our results show that upstream land use must be taken in account. However, herein lays one of the limitations of using this statistical approach as it only takes into account the percentage of land use in the catchment without taking into account geographical position of the pathogen source along hillslopes compared to the hydrological network. In other words, if a pathogen source is located near the hydrological network, then transfer of the bacteria will be more efficient than if the pathogen source is far from the hydrological network (Pachepsky et al. 2011). Moreover, we found that all of the downstream samples (those along the Xe Don River main path; SR) were positive. Therefore, whole catchment-scale approaches are probably not an appropriate sampling strategy. In contrast, the meso-scale catchment scale (Tetzlaff et al. 2007) seems to be more appropriate in the morphopedological context of the Lao landscape (<400 km^2^ in the Nam Xe Done catchment). We therefore propose that future sampling strategies should focus on meso-scale catchments.

Finally, the low estimated abundances of *B. pseudomallei* in the water column led us to use an enrichment step before qPCR analysis, and this should be borne in mind. By using a culturing step (Knappik et al. 2015), we were able to increase the sensitivity of the detection (from 11 to 15 total positive sites; see Table 2 of Knappik et al. (2015)). However, it should be recognized that by using the enrichment step, although we increase the sensitivity of the qPCR method, perhaps a small bias is introduced. However, in the case of our ST7, it allowed us to detect a likely low level of contamination. We therefore recommend using this enrichment step when environmental samples are analyzed for presence or absence of *B. pseudomallei* to minimize the possibility of false negatives.

## Conclusions

SEA is facing rapid changes in land use (Valentin et al. 2008), and knowing how these changes will influence the distribution *B. pseudomallei* and other pathogens in the environment is of utmost importance if we are to reduce the risks to the surrounding populations (Kaestli et al. 2015; Rochelle-Newall et al. 2015). Bearing in mind that the results presented here are from 20 sample sites in one catchment and were collected during 1 month, we show that soil type in the surrounding catchment along with turbidity strongly influences the presence (acrisols and luvisols) or absence (ferralsols) of *B. pseudomallei*. The high presence of acrisols and luvisols and the relatively low presence of ferralsols in SEA, along with the tropical conditions, may well co-act to explain the endemicity of *B. pseudomallei* in SEA and Northern Australia. Conversely, the presence of ferralsols in tropical Africa and South America may also partly explain the low reported presence of *B. pseudomallei* in these regions.

Given the strong apparent links between soil characteristics, water turbidity, and the occurrence of *B. pseudomallei*, actions that raise public awareness and that foster the implementation of land use management practices that reduce the risk of exposure to the pathogen can be proposed in order to reduce the incidence of melioidosis in regions of endemicity. For example, by increasing the risk awareness of farmers who use alluvial terraces for cultures or who use potentially contaminated water for irrigation and by reducing contact with turbid flood waters, the risks of contamination can be reduced.

## References

[CR1] Baker A, Tahani D, Gardiner C, Bristow KL, Greenhill AR, Warner J (2011). Groundwater seeps facilitate exposure to *Burkholderia pseudomallei*. Appl Environ Microbiol.

[CR2] Baker AL, Ezzahir J, Gardiner C, Shipton W, Warner JM (2015). Environmental attributes influencing the distribution of *Burkholderia pseudomallei* in Northern Australia. PLoS ONE.

[CR3] Benzecri JP (1973). L'analyse des données.

[CR4] Bourrié G, Trolard F, Génin JMR, Jaffrezic A, Maître V, Abdelmoula M (1999). Iron, control by equilibria between hydroxy-green rusts and solutions in hydromorphic soils. Geochim Cosmochim Acta.

[CR5] Brereton RG, Lloyd GR (2014). Partial least discriminant analysis: taking the magic away. J Chemom.

[CR6] Buisson Y, Rattanavong S, Keoluangkhot V, Vongphayloth K, Manivanh L, Phetsouvanh R, Pierret A, Maeght J-L, Wuthiekanun V, Newton P, Dance DB, Dujardin J-P, Lefait-Robin R, Apiwathnasorn C, Morand S (2015). Melioidosis in Laos. Socio-ecological dimensions of infectious diseases in Southeast Asia.

[CR7] Cailliez F, Pages JP (1976). Introduction à l'analyse des données.

[CR8] Causse J, Billen G, Garnier J, Henri-des-Tureaux T, Olasa X, Thammahacksa C, Latsachak KO, Soulileuth B, Sengtaheuanghoung O, Rochelle-Newall E, Ribolzi O (2015). Field and modelling studies of *Escherichia coli* loads in tropical streams of montane agro-ecosystems. J Hydro Environ Res.

[CR9] Cheng AC, Currie BJ (2005). Melioidosis: epidemiology, pathophysiology, and management. Clin Microbiol Rev.

[CR10] Collins B (2010) Partial Least Squares Regression, LPAC group meeting

[CR11] Corkeron M, Loehr S, Norton R, Nelson P (2010). Melioidosis case clusters in a tropical urban setting: association with soil type and geomorphology, 19th world congress of soil science.

[CR12] Currie BJ, Mayo M, Anstey NM, Donohoe P, Haase A, Kemp DJ (2001). A cluster of melioidosis cases from an endemic region is clonal and is linked to the water supply using molecular typing of *Burkholderia pseudomallei* isolates. AmJTrop Med Hyg.

[CR13] Currie BJ, Dance DAB, Cheng AC (2008). The global distribution of *Burkholderia pseudomallei* and melioidosis: an update. Trans R Soc Trop Med Hyg.

[CR14] Dance DA (1991). Melioidosis: the tip of the iceberg?. Clin Microbiol Rev.

[CR15] Ding LJ, Su JQ, Xu HJ, Jia ZJ, Zhu YG (2015). Long-term nitrogen fertilization of paddy soil shifts iron-reducing microbial community revealed by RNA-^13^C-acetate probing coupled with pyrosequencing. ISME J.

[CR16] Draper ADK, Mayo M, Harrington G, Karp D, Yinfoo D, Ward L, Haslem A, Currie BJ, Kaestli M (2010). Association of the Melioidosis agent *Burkholderia pseudomallei* with water parameters in rural water supplies in Northern Australia. Appl Environ Microbiol.

[CR17] FAO (1998). World reference base for soil resources.

[CR18] Grunberger O (2015). Dynamiques salines des sols des milieux arides et semi-arides (HDR; in French) University of Montpellier II.

[CR19] Guerinot ML (1994). Microbial iron transport. Annu Rev Microbiol.

[CR20] Hervé M (2013) La PLSDA : Pourquoi, comment ? (in French, consulted 18/03/2015), http://www.maximeherve.com/r-statistiques/divers

[CR21] Hori T, Aoyagi T, Itoh H, Narihiro T, Oikawa A, Suzuki K, Ogata A, Friedrich MW, Conrad R, Kamagata Y (2015). Isolation of microorganisms involved in reduction of crystalline iron(III) oxides in natural environments. Front Microbiol.

[CR22] Inglis TJJ (2010). Sinister soils and risky rhizospheres: the ecology of melioidosis and other soil borne infections, 19th world congress of soil science.

[CR23] Kaestli M, Harrington G, Mayo M, Chatfield MD, Harrington I, Hill A, Munksgaard N, Gibb K, Currie BJ (2015). What drives the occurrence of the melioidosis bacterium *Burkholderia pseudomallei* in domestic gardens?. PLoS Negl Trop Dis.

[CR24] Kato S, Nakamura K, Toki T, J-i I, Tsunogai U, Hirota A, Ohkuma M, Yamagishi A (2012). Iron-based microbial ecosystem on and below the seafloor: a case study of hydrothermal fields of the Southern Mariana trough. Front Microbiol.

[CR25] Kirk G (2004). The biogeochemistry of submerged soils.

[CR26] Knappik M, Dance DAB, Rattanavong S, Pierret A, Ribolzi O, Davong V, Silisouk J, Vongsouvath M, Newton PN, Dittrich S (2015). Evaluation of molecular methods to improve the detection of *Burkholderia pseudomallei* in soil and water samples from Laos. Appl Environ Microbiol.

[CR27] Kvitko BH, Goodyear A, Propst KL, Dow SW, Schweizer HP (2012). *Burkholderia pseudomallei* known siderophores and hemin uptake are dispensable for lethal murine melioidosis. PLoS Negl Trop Dis.

[CR28] Limmathurotsakul D, Wuthiekanun V, Chantratita N, Wongsuvan G, Amornchai P, Day NPJ, Peacock SJ (2010). *Burkholderia pseudomallei* is spatially distributed in soil in Northeast Thailand. PLoS Negl Trop Dis.

[CR29] Limmathurotsakul D, Wongsuvan G, Aanensen D, Ngamwilai S, Saiprom N, Rongkard P, Thaipadungpanit J, Kanoksil M, Chantratita N, Day NPJ, Peacock SJ (2014). Melioidosis caused by *Burkholderia pseudomallei* in drinking water, Thailand, 2012. Emerg Infect Dis.

[CR30] Mathew A, Eberl L, Carlier AL (2014). A novel siderophore-independent strategy of iron uptake in the genus *Burkholderia*. Mol Microbiol.

[CR31] Melton ED, Swanner ED, Behrens S, Schmidt C, Kappler A (2014). The interplay of microbially mediated and abiotic reactions in the biogeochemical Fe cycle. Nat Rev Microbiol.

[CR32] Neilands JB (1995). Siderophores: structure and function of microbial iron transport compounds. J Biol Chem.

[CR33] Ngauy V, Lemeshev Y, Sadkowski L, Crawford G (2005). Cutaneous melioidosis in a man who was taken as a prisoner of war by the Japanese during World War II. J Clin Microbiol.

[CR34] Novak RT, Glass MB, Gee JE, Gal D, Mayo MJ, Currie BJ, Wilkins PP (2006). Development and evaluation of a real-time PCR assay targeting the type III secretion system of *Burkholderia pseudomallei*. J Clin Microbiol.

[CR35] Pachepsky Y, Shelton DR, McLain JET, Patel J, Mandrell RE, Sparks DL (2011). Irrigation waters as a source of pathogenic microorganisms in produce: a review. Advances in agronomy. Advances in agronomy.

[CR36] Pages JP, Cailliez F, Escoufier Y (1979). Analyse factorielle : un peu d’histoire et de géométrie (in French). Revue de statistique appliquée.

[CR37] Palasatien S, Lertsirivorakul R, Royros P, Wongratanacheewin S, Sermswan RW (2008). Soil physicochemical properties related to the presence of *Burkholderia pseudomallei*. Trans R Soc Trop Med Hyg.

[CR38] Rattanavong S, Wuthiekanun V, Langla S, Amornchai P, Sirisouk J, Phetsouvanh R, Moore CE, Peacock SJ, Buisson Y, Newton PN (2011). Randomized soil survey of the distribution of *Burkholderia pseudomallei* in rice fields in Laos. Appl Environ Microbiol.

[CR39] Ribolzi O, Evrard E, Huon S, Rochelle-Newall E, Henri-des-Tureaux T, Silvera N, Thammahacksac C, Sengtaheuanghoung O (2015). Use of fallout radionuclides (^7^Be, ^210^Pb) to estimate resuspension of *Escherichia coli* from streambed sediments during floods in a tropical montane catchment. Environ Sci Pollut Res.

[CR40] Rochelle-Newall EJ, Nguyen TMH, Le TPQ, Sengtaheuanghoung O, Ribolzi O (2015). A short review of faecal indicator bacteria in tropical aquatic ecosystems: knowledge gaps and future directions. Front Microbiol: Aquatic microbiology.

[CR41] Rodionov DA, Dubchak IL, Arkin AP, Alm EJ, Gelfand MS (2005). Dissimilatory metabolism of nitrogen oxides in bacteria: comparative reconstruction of transcriptional networks. Plos Computational Biology.

[CR42] Saejiew A, Grunberger O, Arunin S, Favre Boivin F, Tessier DPB, (2004). Critical coagulation concentration of paddy soil clays in sodium-ferrous iron electrolyte. Soil Sci Soc Am J.

[CR43] Sermswan RW, Wongratanacheewin S, Trakulsomboon S, Thamlikitkul V (2001). Ribotyping of *Burkholderia pseudomallei* from clinical and soil isolates in Thailand. Acta Trop.

[CR44] Shrestha B, Babel MS, Maskey S, van Griensven A, Uhlenbrook S, Green A, Akkharath I (2013). Impact of climate change on sediment yield in the Mekong River basin: a case study of the Nam Ou basin, Lao PDR. Hydrol Earth Syst Sci.

[CR45] Slide RC, Ochiai H (2013) Landslides: processes, prediction, and land use. Water Resources Monograph Series, 18. AGU

[CR46] Stoops G, Eswaran H (1985). Morphological characteristics of wet soils, wetland soils: characterization, classification and utilization.

[CR47] Stumm W, Sulzberger B (1992). The cycling of iron in natural environments: considerations based on laboratory studies of heterogeneous redox processes. Geochim Cosmochim Acta.

[CR48] Su HP, Chan TC, Chang CC (2011). Typhoon-related leptospirosis and melioidosis, Taiwan, 2009. Emerg Infect Dis.

[CR49] Supparattanapan S, Saenjan P, Quantin C, Meaght J-L, Grunberger O (2009). Salinity and organic amendment effects on methane emission from a rain-fed saline paddy field. Soil Sci Plant Nutr.

[CR50] Szymanska E, Saccenti E, Smilde AK, Westerhuis JA (2012). Double-check: validation of diagnostic statistics for PLS-DA models in metabolomics studies. Metabolomics.

[CR51] Tetzlaff D, Malcolm IA, Soulsby C (2007). Influence of forestry, environmental change and climatic variability on the hydrology, hydrochemistry and residence times of upland catchments. J Hydrol.

[CR52] Thioulouse J, Chessel D, Dolédec S, Olivier JM (1997). ADE-4: a multivariate analysis and graphical display software. Stat Comput.

[CR53] Valentin C, Agus F, Alamban R, Boosaner A, Bricquet JP, Chaplot V, de Guzman T, de Rouw A, Janeau JL, Orange D, Phachomphonh K, Phai DD, Podwojewski P, Ribolzi O, Silvera N, Subagyono K, Thiebaux JP, Toan TD, Vadari T (2008). Runoff and sediment losses from 27 upland catchments in Southeast Asia: impact of rapid land use changes and conservation practices. Agric Ecosyst Environ.

[CR54] Vongphayloth K, Rattanavong S, Moore CE, Phetsouvanh R, Wuthiekanun V, Sengdouangphachanh A, Phouminh P, Newton PN, Buisson Y (2012). *Burkholderia pseudomallei* detection in surface water in southern Laos using Moore’s swabs. AmJTrop Med Hyg.

[CR55] Westerhuis J, Hoefsloot HJ, Smit S, Vis D, Smilde A, van Velzen EJ, van Duijnhoven JM, van Dorsten F (2008). Assessment of PLSDA cross validation. Metabolomics.

[CR56] Whitby PW, VanWagoner TM, Springer JM, Morton DJ, Seale TW, Stull TL (2006). *Burkholderia cenocepacia* utilizes ferritin as an iron source. J Med Microbiol.

[CR57] Wuthiekanun V, Limmathurotsakul D, Chantratita N, Feil EJ, Day NPJ, Peacock SJ (2009). *Burkholderia pseudomallei* is genetically diverse in agricultural land in Northeast Thailand. PLoS Negl Trop Dis.

